# Postpartum-Acquired Hemophilia A Presenting as Hemoperitoneum: A Case Report

**DOI:** 10.7759/cureus.11817

**Published:** 2020-12-01

**Authors:** Khalid Azam, Zainab Batool, Ayesha Malik, Manahil Chaudhry, Mohammad Abdullah

**Affiliations:** 1 Medicine, Combined Military Hospital Lahore Medical College and Institute of Dentistry, Lahore, PAK; 2 Medicine, Hameed Latif Hospital, Lahore, PAK

**Keywords:** acquired hemophilia a, activated partial thromboplastin time, factor viii inhibitor, hemoperitoneum, bleeding diathesis

## Abstract

Acquired hemophilia A (AHA) is a bleeding diathesis caused by the production of autoantibodies to factor VIII (FVIII). It manifests as an isolated deranged activated partial thromboplastin time (aPTT) indicating a defect in the intrinsic coagulation pathway. Herein, we report a case of a 26-year-old woman who presented with hemoperitoneum in the postpartum period following a lower segment Caesarean section (LSCS). AHA carries significant mortality if it remains undiagnosed, and early recognition and measures to eradicate the acquired inhibitors are the mainstays of its management.

## Introduction

Acquired hemophilia A (AHA) is a bleeding disorder with a prevalence of approximately one to three cases per million people per year [1] and affects both genders [2]. It has a biphasic age distribution [3], with a small peak between 20-30 years, usually in postpartum women, and a larger peak in patients aged 68-80 years [4]. It can be catastrophic in a postpartum setting, where it is not only associated with bleeding complications in the mother but, due to transplacental transfer of immunoglobulin G (IgG) antibodies, can also cause bleeding complications in neonates [5]. The coagulation disorder is caused by the development of autoantibodies or inhibitors, directed against the plasma coagulation factor VIII (FVIII), which eventually alters the coagulation cascade and leads to ineffective clotting. While no underlying cause is identified in almost half of the patients, some common conditions associated with AHA are autoimmune diseases, malignancies, dermatological disorders, pregnancy, and drugs [4].

As per the European Acquired Hemophilia Registry (EACH2), the presence of an unexplained isolated prolonged activated partial thromboplastin time (aPTT) in a peripartum woman with no prior personal or family history of bleeding disorders should be considered as AHA until proven otherwise [6]. To the best of our knowledge, this is the first case of an acquired coagulation defect diagnosed in a 26-year-old woman in the postpartum period to be reported regionally.

## Case presentation

A 26-year-old multipara at 38 weeks + two days of her second pregnancy presented with labor pains to the gynecology and obstetrics emergency department. Her pregnancy had been uneventful throughout and, at the time of presentation, she was vitally stable with a viable fetus. An emergency lower segment Caesarean section (LSCS) was planned due to the failure of the progression of labor, and a healthy baby girl was delivered. Prior to the pregnancy, the patient had regular menstrual cycles with no history of menorrhagia. There was also no history of easy bruising or skin rashes, joint pains, or fevers. Her past medical history was unremarkable. There were no diseases in her family including blood disorders.

**Figure 1:**
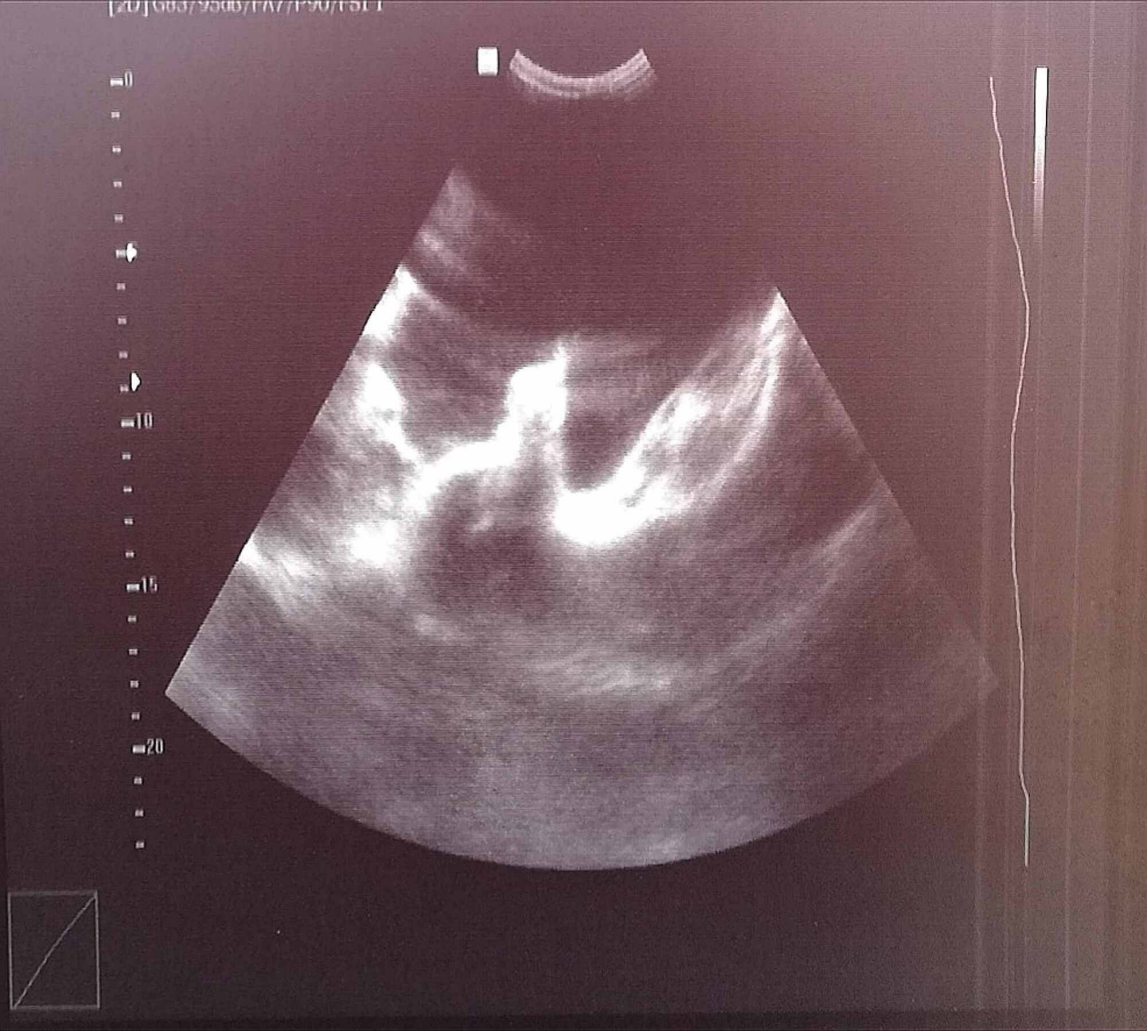
Ultrasound image showing free fluid in the pelvic cavity

On her first postoperative day, the patient complained of abdominal pain. Her vitals showed a hemodynamically stable state; however, on general physical examination, she appeared pale. There was ecchymosis around the surgical site and her bandage was completely soaked with blood. Abdominal examination revealed tenderness in the lower abdomen with a dull percussion note in the flanks. The cardiovascular, respiratory, and neurological examinations were unremarkable. A bedside ultrasound revealed free fluid in the abdominal cavity (Figure 1), which was aspirated under ultrasound guidance, and the aspirate was found to be hemorrhagic. Due to a suspicion of hemoperitoneum, an emergency exploratory laparotomy was performed, and she received a transfusion of blood products for hemostasis concurrently. An urgent preoperative complete blood picture revealed a hemoglobin (Hb) level of 4.5 g/dl. Intraoperative findings confirmed our suspicion.

She was shifted to the intensive care unit (ICU) after the procedure and her entire baseline profile was repeated, which showed a sudden drop in the Hb level from 11.5 g/dl (preoperative) to 4.5 g/dl, a total leukocyte count of 7.0 x 10^9^/L, and a platelet count of 185 x 10^9^/L. Coagulation parameters showed a normal prothrombin time (PT) and international normalized ratio (INR), but an isolated deranged aPTT of 90 seconds (reference range: 0-32 seconds), which was found to be elevated on serial measurements. Further workup was done to evaluate the cause of isolated elevated aPTT levels. Laboratory investigations revealed normal lupus anticoagulant titer, normal bleeding time, normal von Willebrand factor antigen, and normal anti-phospholipid IgM and IgG.

Based on the sudden fall in her Hb, massive abdominal hemorrhage, an isolated increase in the aPTT, and negative results of the aforementioned factors, a diagnosis of acquired hemophilia was considered, and her serum was then sent for mixing studies. She had a normal clotting time at 10 minutes but a delayed clotting time at 120 minutes and her FVIII level was 1%. An FVIII coagulation inhibitor assay (qualitative) was done, which was positive for inhibitors. She was then started on high-dose steroid therapy (oral prednisone 60 mg/day). She responded well to treatment, and her last aPTT before being discharged from the hospital was 46 seconds. There were no further complaints of any bleeding episodes after commencing steroid therapy. She was called for a follow-up, and a steroid-sparing drug, azathioprine, was added to her treatment regimen, and steroids were tapered gradually.

## Discussion

Pregnancy-associated AHA represents 7-11% of the entire disease burden, with patients commonly presenting within one to four months of delivery with variable presentations of bleeding [1]. A review of the last 10 years of reported postpartum AHA cases showed that the ages of patients ranged between 19 years [7] to 40 years [8]. This indicates that AHA related to pregnancy can occur in any woman of childbearing age. The mode of presentation remains similar even with the variation in the days of occurrence. Our patient reported the occurrence of a massive hemorrhagic disease on the very first day post-LSCS, while Spencer et al. [9] have reported the occurrence of massive hematomas in a primigravida five weeks post-LSCS. In another study, Lee et al. [8] have reported a vaginal bleed five days after the LSCS, and the development of large hematomas 65 days after the LSCS. Up to 9% of cases present with retroperitoneal bleeds and the bleeding pattern in AHA differs from congenital hemophilia, where mostly joint bleeds are seen [10].

The diagnostic parameter of AHA is the coagulation profile indicating a defect in the intrinsic pathway and, as mentioned before, the hallmark is an isolated deranged aPTT. A study by Kose et al. [7] has reported a case of postpartum AHA with an initial aPTT value of >120 seconds, which is the highest to be reported yet. Our case presented with an aPTT value of 90 seconds, which raised the suspicion of acquired hemophilia, which we confirmed by mixing studies. Guidelines recommend that the diagnosis of AHA be considered whenever a sudden onset of bleeding occurs along with unexplained prolonged aPTT [11]. Standard guidelines recommend going for either a Bethesda assay or an anti-FVIII enzyme-linked immunosorbent assay (ELISA). Due to the unavailability of the former testing method in our setup, we proceeded to confirm the diagnosis based on the ELISA method. The patient's aPTT was serially followed while she was on the recommended treatment, and she responded well. The severity of hemophilia is currently classified based on plasma levels of FVIII activity: severe if <1%, moderate if between 1 and 5%, and mild if >5 and <40% of normal [11]. The former severe form of presentation was seen in our case.

The initiation of treatment at an early stage, to eradicate inhibitors, prevents morbidity and mortality in such patients. Based on current guidelines, patients should be treated with corticosteroid therapy, either prednisolone or prednisone at a dose of 1 mg/kg/day per oral (PO) for four to six weeks, followed by tapering withdrawal. Corticosteroids should be used either alone or in combination with a cytotoxic agent like cyclophosphamide (1.5-2 mg/kg/day PO for a maximum of six weeks) or mycophenolate mofetil (1 g/day for one week, followed by 2 g/day). In cases where first-line immunosuppressive therapy fails or is contraindicated, rituximab (375 mg/m^2^ weekly for a maximum of four cycles) is recommended [10,12]. Following eradication therapy, follow-ups after a complete sustained response using serial aPTT and monitoring of FVIII monthly during the first six months, every two to three months up to 12 months, and every six months during the second year and beyond is recommended. Once in remission, thromboprophylaxis using either antiplatelet drugs or oral anticoagulants is also recommended [12].

## Conclusions

Hemorrhagic disease associated with pregnancy, with no personal or family history of bleeding disorders, and an isolated deranged aPTT should raise a high clinical suspicion of AHA. This presentation warrants a prompt diagnosis and early initiation of treatment as the condition can be potentially fatal.
